# Thermotolerance of tomato plants grafted onto wild relative rootstocks

**DOI:** 10.3389/fpls.2023.1252456

**Published:** 2023-11-20

**Authors:** Chungkeun Lee, Joshua T. Harvey, Asmita Nagila, Kuan Qin, Daniel I. Leskovar

**Affiliations:** Texas A&M AgriLife Research and Extension Center, Texas A&M University, Uvalde, TX, United States

**Keywords:** *S. peruvianum*, *S. pennellii*, Maxifort, antioxidant, abscisic acid, FRAP, scion, growth chamber

## Abstract

Heat stress is a major environmental constraint limiting tomato production. Tomato wild relatives *Solanum pennellii* and *S. peruvianum* are known for their drought tolerance but their heat stress responses have been less investigated, especially when used as rootstocks for grafting. This study aimed to evaluate the physiological and biochemical heat stress responses of tomato seedlings grafted onto a commercial ‘Maxifort’ and wild relative *S. pennellii* and *S. peruvianum* rootstocks. ‘Celebrity’ and ‘Arkansas Traveler’ tomato scion cultivars, previously characterized as heat-tolerant and heat-sensitive, respectively, were grafted onto the rootstocks or self-grafted as controls. Grafted seedlings were transplanted into 10-cm pots and placed in growth chambers set at high (38/30°C, day/night) and optimal (26/19°C) temperatures for 21 days during the vegetative stage. Under heat stress, *S. peruvianum*-grafted tomato seedlings had an increased leaf proline content and total non-enzymatic antioxidant capacity in both leaves and roots. Additionally, *S. peruvianum*-grafted plants showed more heat-tolerant responses, evidenced by their increase in multiple leaf antioxidant enzyme activities (superoxide dismutase, catalase and peroxidase) compared to self-grafted and ‘Maxifort’-grafted plants. *S. pennellii*-grafted plants had similar or higher activities in all antioxidant enzymes than other treatments at optimal temperature conditions but significantly lower activities under heat stress conditions, an indication of heat sensitivity. Both *S. pennellii* and *S. peruvianum*-grafted plants had higher leaf chlorophyll content, chlorophyll fluorescence and net photosynthetic rate under heat stress, while their plant growth was significantly lower than self-grafted and ‘Maxifort’-grafted plants possibly from graft incompatibility. Root abscisic acid (ABA) contents were higher in ‘Maxifort’ and *S. peruvianum* rootstocks, but no ABA-induced antioxidant activities were detected in either leaves or roots. In conclusion, the wild relative rootstock *S. peruvianum* was effective in enhancing the thermotolerance of scion tomato seedlings, showing potential as a breeding material for the introgression of heat-tolerant traits in interspecific tomato rootstocks.

## 1 Introduction

Due to the rapidly changing global climate, high temperature stress has become one of the major threats to the productivity of field-cultivated vegetable crops worldwide. Tomato (*Solanum lycopersicum* L.), which is the second most produced vegetable in the world, has also been facing such challenges in hot climatic regions. Tomato plants have a narrow temperature range for their optimal growth and development, 25-30°C during daytime and 20°C at night (Camejo et al., 2005), and exhibit heat stress symptoms when ambient temperatures exceed 35°C (Wahid et al., 2007).

Heat stress induces excessive production of reactive oxygen species (ROS), and the imbalance between the produced ROS and their detoxification causes oxidative stress (Tripathy and Oelmuller, 2012). The accumulation of oxidative damage in chlorophyll, cell membrane, protein and DNA and RNA molecules causes adverse effects on plant metabolism such as reduced photosynthesis and disruption of reproductive processes during anthesis (Mittler, 2002; Wahid et al., 2007). The photosystem II (PSII), a highly thermolabile photosynthetic component, is particularly susceptible to damage from heat stress, which results in reduced photochemical efficiency with increased non-photochemical quenching (Bukhov et al., 1999; Baker, 2008).

In response to heat stress, plants accumulate compatible osmolytes such as proline, γ-4-aminobutyric acid (GABA) and sugars and produce ROS scavenging enzymes including superoxide dismutase (SOD), catalase (CAT), guaiacol peroxidase (POD), ascorbate peroxidase (APX) and glutathione reductase (GR) (Bohnert et al., 1995; Gill and Tuteja, 2010). Plants also regulate the stress hormone abscisic acid (ABA) to induce antioxidant activities and heat shock proteins, which is mediated by ROS signaling (Islam et al., 2018).

Grafting allows the conjugation of desirable crop phenotypes from scion and rootstock varieties. It is widely used for improving vegetable production and quality, especially in *Solanaceae* and *Cucurbitaceae* crops (Martínez-Ballesta et al., 2010). Not only can grafting alleviate soil-borne disease and enhance nutrient and water absorption but can also increase tolerance to abiotic stresses such as drought, salinity and high temperature (Schwarz et al., 2010). Bidirectional communication between scion and rootstock enables grafted plants to transfer stress tolerance traits from the rootstock to the scion through the transport of stress-related metabolites, peptides and nucleic acids (Estan et al., 2009; Asins et al., 2010; Albacete et al., 2015). Several studies have reported that when using heat-tolerant rootstocks there were physiological and biochemical enhancements of the scion thermotolerance in grafted tomato seedlings (Rivero et al., 2003a; Rivero et al., 2003b; Abdelmageed and Gruda, 2009).

Commercial tomato varieties have narrow genetic diversity as a result of intensive domestication (Williams and Clair, 1993). Wild tomato species, on the other hand, have greater genetic variation, and grafting onto these rootstocks could enhance the scion performance under heat stress conditions through potentially greater availability of heat-tolerant genotypes that existed before domestication (Rick, 1984; Peralta et al., 2008). *Solanum pennellii* and *S. peruvianum* are tomato wild relatives endemic to arid Andean regions of central Peru to northern Chile (Rick and Tanksley, 1981; Knapp and Peralta, 2016) and are characterized as drought and salinity tolerant (Tapia et al., 2016; Zaki and Yokoi, 2016; Egea et al., 2018). The abiotic stress tolerance strategy of *S. pennellii* and *S. peruvianum* involves osmotic adjustments by the accumulation of high compatible osmolytes such as proline and carbohydrates and sensitive control of the stomatal behavior (Yu, 1972; Tapia et al., 2016; Egea et al., 2018). In recent years, some studies have examined the thermotolerance of *S. pennellii* and *S. peruvianum*, showing that heat-stressed wild tomato species had high photosynthetic capacity and photosystem efficiency during the seedling stage (Zhou et al., 2018) and high pollen number and viability during the reproductive stage (Driedonks et al., 2018). Their superior tolerance to heat stress could potentially provide wild species materials to be used as rootstocks for grafted tomato production in regions with elevated temperatures. However, evaluations of this nature have been reported less frequently.

In this study, we investigated the heat tolerance traits of *S. pennellii* and *S. peruvianum* rootstocks and their physiological and biochemical responses when grafted into two commercial tomato scion cultivars differing in thermotolerance. This research explored the leaf and root enzymatic and non-enzymatic antioxidant capacity and established their correlation with physiological and hormonal stress responses. A previous investigation from our group demonstrated the potential of *S. pennellii* and *S. peruvianum* as rootstocks to enhance the drought tolerance of grafted tomato plants (Alves et al., 2021). Also, according to Mittler (2006), plants could share some common defense mechanisms against drought and heat stress conditions. Therefore, we hypothesized that grafting tomato seedlings onto *S. pennellii* and *S. peruvianum* rootstocks would enhance the thermotolerance by using heat and drought tolerance strategies of wild tomato relatives such as high photosynthetic ability and antioxidant capacity, respectively.

## 2 Materials and methods

### 2.1 Plant materials and growth conditions

Tomato cultivars ‘Celebrity’ (Clifton Seed Company, Faison, NC, USA) and ‘Arkansas Traveler’ (Seeds ‘n Such, Graniteville, SC, USA) were selected from the thermotolerance screening by Bhattarai et al. (2021) which identified them as heat-tolerant and sensitive cultivars, respectively. The scion cultivars were grafted onto interspecific hybrid rootstock ‘Maxifort’ (*Solanum lycopersicum* L. × *Solanum habrochaites* S. Knapp & D.M. Spooner; Johnny’s Selected Seeds, Fairfield, ME, USA) and wild relative rootstocks, *S. pennellii* (LA0716) and *S. peruvianum* (PI128659). The wild tomato accessions were obtained from the UC Davis/C.M. Rick Tomato Genetics Resource Center, maintained by the Department of Plant Sciences, University of California, Davis, CA, USA.

Seeds of two scion cultivars and three rootstock species were sown in 200-cell polystyrene trays (2.5 × 2.5 × 7.6 cm, 32 cm^3^ cell volume; TR200A model, Speedling, Ruskin, FL, USA) filled with growing media (90% sphagnum peat moss, 10% perlite and vermiculite; LM-CB, Lambert Peat Moss Inc., Quebec, Canada), while *S. peruvianum*, *S. pennellii* and ‘Maxifort’ were seeded 28 days, 21 days and 7 days earlier, respectively, than scion cultivars to synchronize their stem diameter for grafting. The seedlings were grown in a greenhouse with a supplementary fluorescent light system (50 µmol m^-2^ s^-1^) for 5 weeks after seeding scion cultivars and fertigated once a week with an overhead irrigation system using Peters Professional 20-20-20 fertilizer (100 to 150 mg N L^-1^) (Peters Professional, ICL Specialty Fertilizers, Tel Aviv, Israel) beginning 3 weeks after seeding.

Seedlings were splice-grafted when the average stem diameter reached the target size (2.0 mm) and the graft union was held using a silicone graft clip (Johnny’s Selected Seeds) during a 12-day acclimation period. The grafted plants were healed in a growth chamber (GEN1000, Conviron, Winnipeg, Canada) at 23°C and 95-98% relative humidity (RH) with no light and were slowly introduced to lower RH and dim light beginning on the third day of acclimation. The acclimated plants were additionally hardened off for 7 days in a greenhouse.

The grafted plants were transplanted into square plastic pots (9.5 × 9.5 × 9 cm, 810 cm^3^ volume, BWI Companies, Inc., Nash, TX, USA) filled with the LM-CB growing media inside the growth chamber. High temperature stress (38/30°C, 16/8 h, day/night) was introduced after 5 days of transplant acclimation at 26/19°C, while the temperature control group remained at the same temperature regime. One growth chamber was used for each temperature treatment, with horizontal partitions in each chamber. The RH and light intensity were controlled at 60% and 300 µmol m^-2^ s^-1^ PAR, respectively, and the pots were irrigated daily with tap water to saturation with a weekly fertigation of 200 mg N L^-1^ using 20-20-20 fertilizer.

Our experiment followed a completely randomized factorial design with three treatment factors, including temperature, scion and rootstock. Each treatment combination had five replicates (i.e. individual plants). Morphological, physiological and biochemical measurements were taken 3 weeks after the stress period.

### 2.2 Growth and chlorophyll content measurements

Plant stem diameter (mm) and shoot fresh weight (g; FW) were measured; stem diameter was measured above the graft union using a digital caliper to evaluate the effect of rootstock on the scion. SPAD was measured to estimate the leaf chlorophyll content per unit area using a portable chlorophyll meter (SPAD-502 Plus, Konica Minolta, Tokyo, Japan). The average value of five readings per plant were taken from fully expanded mature leaves.

### 2.3 Chlorophyll fluorescence determination

Chlorophyll fluorescence (F_v_/F_m_) was measured to assess the maximum quantum yield efficiency of PSII in leaves with a portable chlorophyll fluorometer (Op30p; Opti-Sciences Inc., Hudson, NH, USA). The second fully expanded leaves from the shoot apex were dark-adapted for 30 min using a dark clip before the measurement, and the minimum (F_o_) and maximum (F_m_) fluorescence were recorded by the fluorometer.

### 2.4 Gas exchange measurements

Net photosynthetic rate (μmol CO_2_ m^-2^ s^-1^), transpiration rate (mmol H_2_O m^-2^ s^-1^) and stomatal conductance (mol H_2_O m^-2^ s^-1^) were measured using a portable photosynthesis system (LI-6400 XT, LICOR Biosciences, NE, USA). The measurements were taken from 9 a.m. to 2 p.m. on the fully expanded mature leaves. The chamber environment was controlled by the sensor head under the following conditions: light intensity, 1000 μmol m^−2^ s^−1^ (LED, 10% blue 90% red); CO_2_ concentration, 400 ppm; air flow rate, 400 μmol s^-1^.

### 2.5 Identification of reactive oxygen species content

The total reactive oxygen species (ROS) contents of leaves and roots were measured using the methods of Alexou (2013) and Michael et al. (2007). Leaf and root samples (100 mg) were flash-frozen with liquid nitrogen and ground to powder using a mortar and pestle. The powdered samples were homogenized with 100 mg polyvinylpyrrolidone in 1 mL distilled water and centrifuged at 16,000 × *g* for 20 min at 4°C. Eighty microliters of 3 mM luminol solution in dimethyl sulfoxide was added to 400 µL of the supernatants and the absorbance at 380 nm was measured using a UV-vis spectrophotometer (Genesys™ 10S UV-Vis, Thermo Fisher Scientific, Waltham, MA, USA). The absorbance of the solution without luminol was used as a control for each measurement, and the relative values of ROS were used to compare the differences between the treatments.

### 2.6 FRAP assay

The total antioxidant capacity of leaves and roots were estimated using the ferric reducing antioxidant power (FRAP) assay described by Benzie and Strain (1996) with modifications. A 2 mL working solution containing 300 mM acetate buffer (pH 3.6), 10 mM 2,4,6-tri(2-pyridyl)-S-triazine (TPTZ) in 40 mM HCl and 20 mM iron chloride (FeCl_3_) in a ratio of 10:1:1 was added to the 100 µL of supernatant extracted from 100 mg of flash-frozen and powdered samples homogenized with methanol. The antioxidant capacity was determined by measuring the absorbance at 593 nm and the absorbance of the solution without TPTZ was used as a control for each sample.

### 2.7 Proline content determination

The leaf proline contents were measured using a modified method of Bates et al. (1973). Flash-frozen and powdered samples were homogenized with 1.5 mL of 3% sulfosalicylic acid, and the supernatants from centrifugation were added with 0.5 mL of acetic acid and 0.5 mL of ninhydrin reagent (2.5% ninhydrin in 10.44 M acetic acid and 2.4 M phosphoric acid). After boiling the reaction mixture at 95°C for 1 h, 0.8 mL of toluene was added and the absorbance at 520 nm was measured using toluene as a blank. The standard curve was obtained with 1, 10, 50, 100, 150, 200 and 300 μM of D-proline.

### 2.8 Determination of antioxidant enzyme activities

The antioxidant enzyme activities were estimated from leaf and root samples. The flash-frozen and powdered samples (200 mg) were extracted in 1.2 mL of 0.2 M potassium phosphate buffer (pH 7.8) with 0.1 mM EDTA and the supernatants were collected after centrifugation at 16,000 × *g* for 20 min at 4°C.

#### 2.8.1 Superoxide dismutase

The superoxide dismutase (SOD) activity was determined by adding 100 μL of enzyme extract to the 2 mL reaction mixture containing 50 mM phosphate buffer (pH 7.8), 2 mM EDTA, 9.9 mM L-methionine, 55 μM NBT and 0.025% Triton X-100, and the reaction initiated by adding 20 μL of 146 μM riboflavin under LED light for 10 min (Giannopolitis and Ries, 1977). The absorbance was measured at 560 nm and the standard curve was generated with 0.05, 0.1, 0.2, 1 and 5 units of SOD enzyme.

#### 2.8.2 Catalase

The catalase (CAT) activity was measured using a modified method of Hadwan (2018). The enzyme extract (150 μL) added with 300 uL of 10 mM hydrogen peroxide (H_2_O_2_) reacted with the 1.8 mL of a working solution containing 3.48 mM cobalt (II) nitrate hexahydrate, 0.82 mM sodium hexametaphosphate and 96.4 mM sodium bicarbonate for 2 min. The absorbance was measured at 440 nm and distilled water was used instead of enzyme extract for measuring the control absorbance.

#### 2.8.3 Guaiacol peroxidase

The guaiacol peroxidase (POD) activity was measured by adding 0.5 mL of 12 mM H_2_O_2_ into a 2 mL reaction mixture containing 10 mM potassium phosphate buffer (pH 6.1), 96 mM guaiacol and 100 μL of enzyme extract (Castillo et al., 1984). The absorbance was measured at 470 nm for 2 min and the enzyme activity was calculated using the extinction coefficient of guaiacol (26.6 mM^-1^ cm^-1^).

#### 2.8.4 Ascorbate peroxidase

The ascorbate peroxidase (APX) activity was assessed using the method of Nakano and Asada (1981) with modifications. The assay mixture (2 mL) containing 50 mM potassium phosphate buffer (pH 7.0), 0.5 mM ascorbic acid, 1 mM EDTA and 150 μL of enzyme extract reacted with 200 uL of 0.3 mM H_2_O_2_ solution for 20 sec by vortexing. The absorbance was measured at 290 nm before and after the reaction and the enzyme activity was calculated using the extinction coefficient of ascorbic acid (2.8 mM^-1^ cm^-1^).

#### 2.8.5 Glutathione reductase

The glutathione reductase (GR) activity was assayed as described by Smith et al. (1988). The reaction was initiated by adding 0.5 mL of 0.5 mM oxidized glutathione (GSSG) into the 2 mL assay mixture containing 0.75 mM DTNB (5,5-dithiobis [2-nitrobenzoic acid]), 0.1 mM NADPH and 100 μL of enzyme extract. The absorbance was measured at 412 nm for 3 min and the enzyme activity was calculated using the extinction coefficient of TNB (5-thio-2-nitrobenzoic acid) (14.15 mM^-1^ cm^-1^).

#### 2.8.6 Total soluble protein content

The total soluble protein content of leaf and root samples were measured following the method of Bradford (1976). The absorbance at 595 nm was measured after incubating the reaction mixture containing 2 mL Bradford reagent and enzyme extract (leaf, 40 μL; root, 80 μL) for 10 min. The standard curve was obtained by using 0.25, 0.5, 1, 1.5 and 2 mg mL^-1^ of bovine serum albumin.

### 2.9 Abscisic acid content determination

The abscisic acid (ABA) contents of leaf and root samples were determined by using Phytodetek-ABA immunoassay kit (Agdia Inc., Elkhart, IN, USA). The flash-frozen and powdered samples (100 mg) were extracted with extraction buffer (90% methanol containing 0.89 mM sodium diethyldithiocarbamate trihydrate) and methanolic tris buffer (10% methanol containing 50 mM Tris, 1 mM MgCl_2_ and 150 mM NaCl) (Liu et al., 2014). The subsequent analysis was performed according to the manufacturer’s protocol. The absorbance was measured at 405 nm using a microplate spectrophotometer reader (Multiskan™ GO microplate Spectrophotometer, Thermo Scientific, Vantaa, Finland) with a correction wavelength set at 540 nm.

### 2.10 Statistical analysis

All experimental data were subjected to a three-way analysis of variance (ANOVA) to examine the effects of temperature, scion and rootstock varieties using R software version 4.2.0 (R Development Core Team, 2022). Differences between treatment means were compared using Duncan’s multiple range test at *P* = 0.05 level of significance with the ‘agricolae’ package (De Mendiburu, 2014). Pearson’s correlation coefficient method was used to evaluate the relationship between physio-biochemical responses. The analysis of gas exchange data included the time of the measurement as a covariate to remove the time effect on the results.

## 3 Results

### 3.1 Plant growth and leaf chlorophyll content

**Figure 1:**
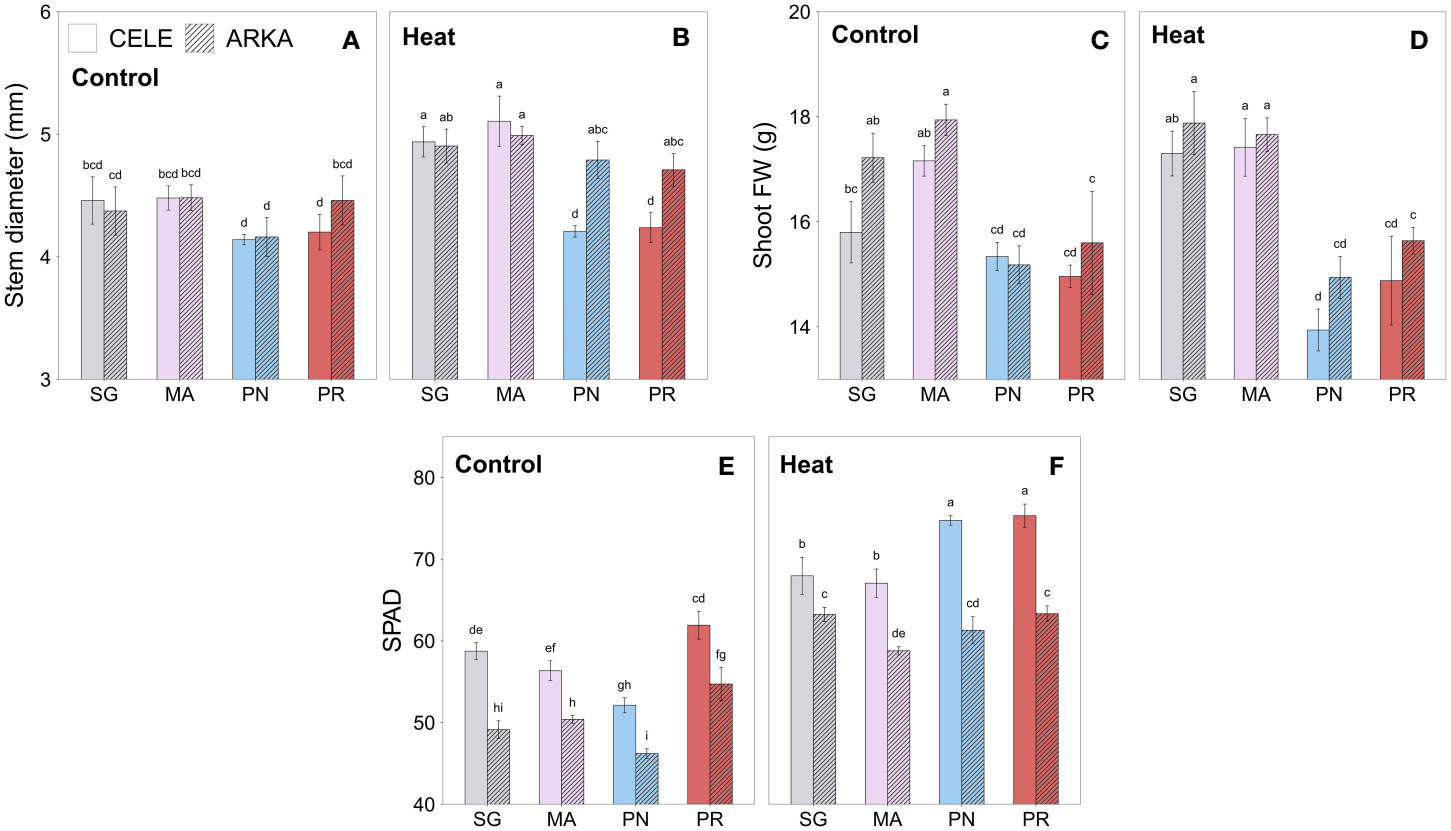
Heat stress responses of stem diameter **(A, B)**, shoot fresh weight **(C, D)** and SPAD **(E, F)** in tomato plants self-grafted or grafted onto ‘Maxifort’, *S. pennellii* and *S. peruvianum* rootstocks. Different letters indicate significant differences among scion-rootstock combinations across the temperature treatments (control, heat) at *P* ≤ 0.05; CELE, ‘Celebrity’; ARKA, ‘Arkansas Traveler’; SG, self-grafted; MA, ‘Maxifort’; PN, *S. pennellii*; PR, *S. peruvianum*.

Heat stress significantly increased the stem diameter of self-grafted (SG) and ‘Maxifort’ (MA)-grafted tomato plants, while *S. peruvianum* (PR)-grafted plants showed no significant differences compared to the controls (**Figures 1A, B**; **Supplementary Table 2**). In *S. pennellii* (PN)-grafted plants, only ‘Arkansas Traveler’ scion treatment groups showed a significant increase in stem diameter by heat stress.

There were no significant differences between control and heat treatment in shoot FW in all grafting combinations (**Figures 1C, D**). Regardless of stress conditions, SG and MA-grafted plants had significantly higher average shoot FW than PN and PR-grafted plants, except for SG ‘Celebrity’ at control temperature (**Figures 1C, D**).

Mean SPAD values increased under heat stress in all grafting combinations with PN-grafted plants showing the highest increase among them (**Figures 1E, F**; **Supplementary Table 2**; **Supplementary Figure 1A, B**). PR-grafted plants had similar or higher SPAD value than other rootstock treatment groups in control conditions when compared within scion cultivars and were still significantly higher than SG and MA-grafted plants under heat stress for ‘Celebrity’ (**Figure 1F**). ‘Celebrity’ scion showed a higher mean SPAD value than ‘Arkansas Traveler’ regardless of the stress conditions when compared within the rootstock treatments.

### 3.2 Maximum quantum efficiency of PSII and gas exchange capacity

**Figure 2:**
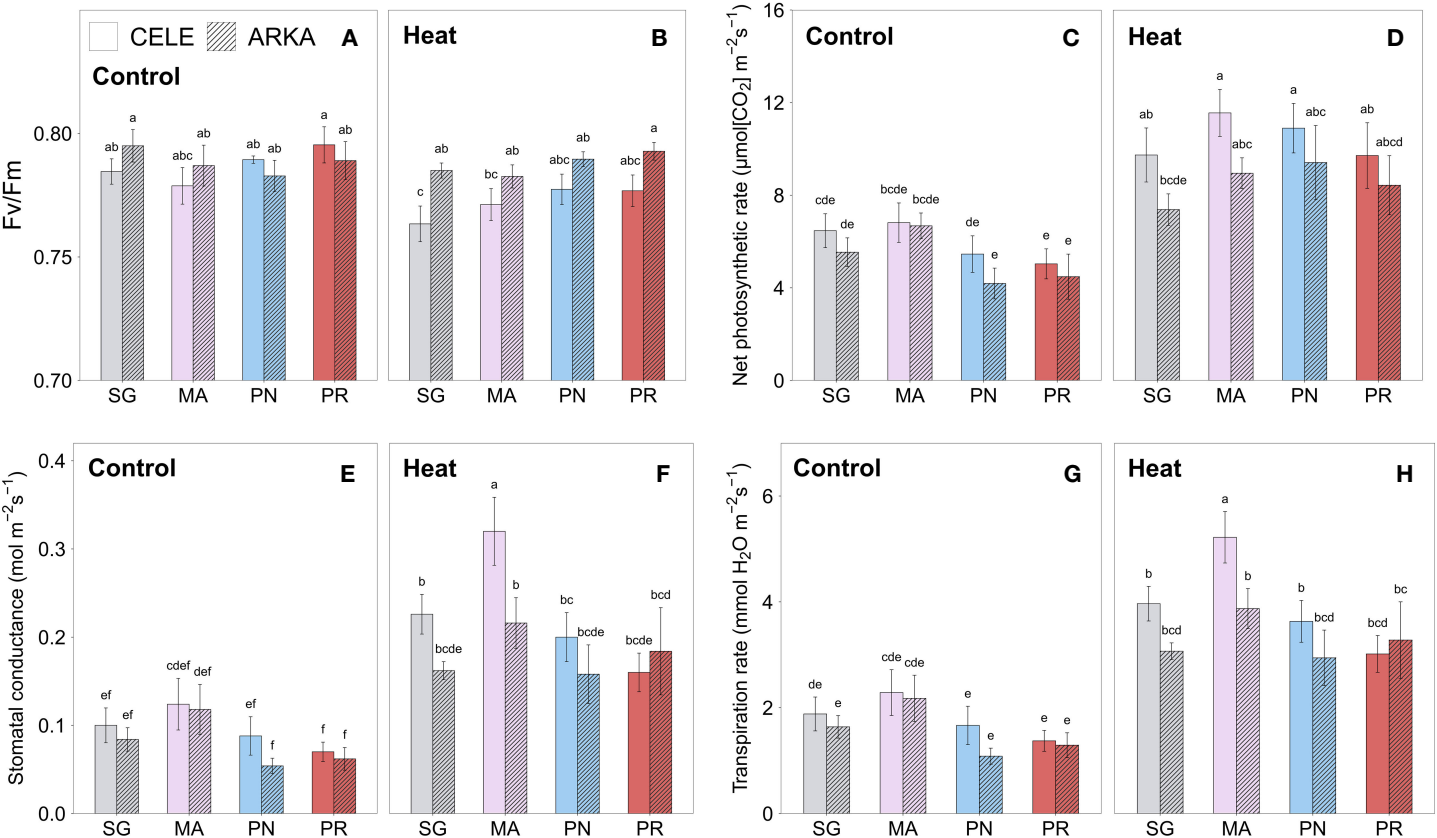
Heat stress responses of chlorophyll fluorescence **(A, B)**, net photosynthetic rate **(C, D)**, stomatal conductance **(E, F)** and transpiration rate **(G, H)** in tomato plants self-grafted or grafted onto ‘Maxifort’, *S. pennellii* and *S. peruvianum* rootstocks. Different letters indicate significant differences among scion-rootstock combinations across the temperature treatments (control, heat) at *P* ≤ 0.05; CELE, ‘Celebrity’; ARKA, ‘Arkansas Traveler’; SG, self-grafted; MA, ‘Maxifort’; PN, *S. pennellii*; PR, *S. peruvianum*.

Chlorophyll fluorescence (F_v_/F_m_), which estimates the maximum quantum yield efficiency of PSII, significantly decreased under heat stress in SG ‘Celebrity’ plants while the rest of the treatment combinations showed no significant differences (**Figures 2A, B**; **Supplementary Table 2**). Significant temperature × cultivar interactions were found with heat stress reducing the F_v_/F_m_ more in ‘Celebrity’ while it decreased less in ‘Arkansas Traveler’ plants (**Supplementary Table 1**), an indication that this cultivar maintained the PSII efficiency.

Heat stress significantly increased net photosynthetic rate (**Figures 2C, D**), stomatal conductance (**Figures 2E, F**) and leaf transpiration rate (**Figures 2G, H**) in all grafting combinations, except for net photosynthetic rate of SG and MA-grafted ‘Arkansas Traveler’ and stomatal conductance of SG ‘Arkansas Traveler’ plants. PN and PR-grafted plants showed a larger increase in net photosynthetic rate than SG and MA-grafted groups (**Supplementary Figures 1A, B**), while MA-grafted ‘Celebrity’ plants showed the greatest increase in stomatal conductance and transpiration rate among all treatment groups, resulting in the highest values under heat stress conditions (**Figures 2F, H**).

### 3.3 ROS concentration, total antioxidant capacity and leaf proline content

**Figure 3:**
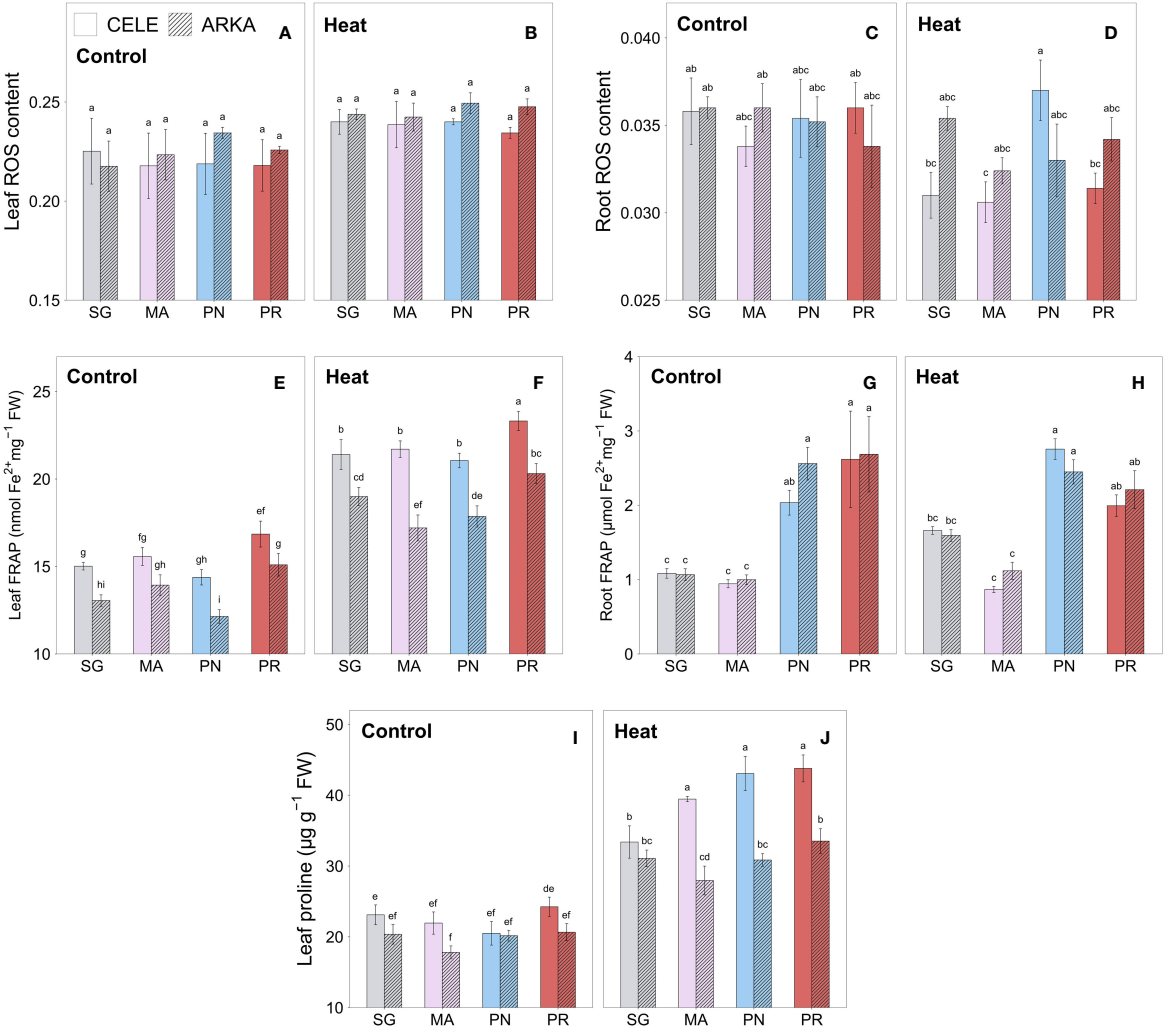
Heat stress responses of leaf **(A, B)** and root **(C, D)** ROS content, leaf **(E, F)** and root **(G, H)** FRAP and leaf proline content **(I, J)** in tomato plants self-grafted or grafted onto ‘Maxifort’, *S. pennellii* and *S. peruvianum* rootstocks. Different letters indicate significant differences among scion-rootstock combinations across the temperature treatments (control, heat) at *P* ≤ 0.05; CELE, ‘Celebrity’; ARKA, ‘Arkansas Traveler’; SG, self-grafted; MA, ‘Maxifort’; PN, *S. pennellii*; PR, *S. peruvianum*.

There were no significant differences in leaf ROS content between all grafting combinations in both control and heat stress conditions, while heat stressed plants had numerically higher values compared to the controls (**Figures 3A, B**). On the other hand, in root ROS, PN-grafted ‘Celebrity’ exhibited a higher value than the other rootstock treatment groups under heat stress, while no significant differences were observed between treatments at control temperature (**Figures 3C, D**). In heat stressed leaves, ‘Celebrity’ had marginally lower ROS than ‘Arkansas Traveler’ (*P* = 0.083), on average.

The total non-enzymatic antioxidant capacity measured using the FRAP assay showed a significant increase in all grafting combinations by heat stress in leaf samples (**Supplementary Table 2**; **Supplementary Figures 1C, D**), while the PR-grafted plants had similar or higher value regardless of stress conditions within each scion (**Figures 3E, F**). ‘Celebrity’ cultivar had higher leaf total antioxidant capacity on average than ‘Arkansas Traveler’, except for MA-grafted plants in the control treatment. In root samples, both PN and PR-grafted plants demonstrated higher total antioxidant capacity compared to SG and MA-grafted plants in control and MA-grafted plants in heat stress conditions, while no significant heat stress responses were observed within the rootstock treatments (**Figures 3G, H**).

**Figures 3I, J** show that all grafting treatment groups had increased leaf proline content under heat stress conditions compared to the controls (**Supplementary Table 2**; **Supplementary Figures 1A, B**). Under heat stress conditions, the proline contents in PR-grafted plants were significantly higher than that in SG and MA in ‘Celebrity’ and ‘Arkansas Traveler’, respectively, within the scions. There were no significant differences between the grafting combinations in the control treatment. ‘Celebrity’ plants grafted onto MA, PN and PR demonstrated a greater increase in leaf proline content than those with ‘Arkansas Traveler’ when exposed to heat stress, resulting in significantly higher values.

### 3.4 Activities of antioxidant enzymes

**Figure 4:**
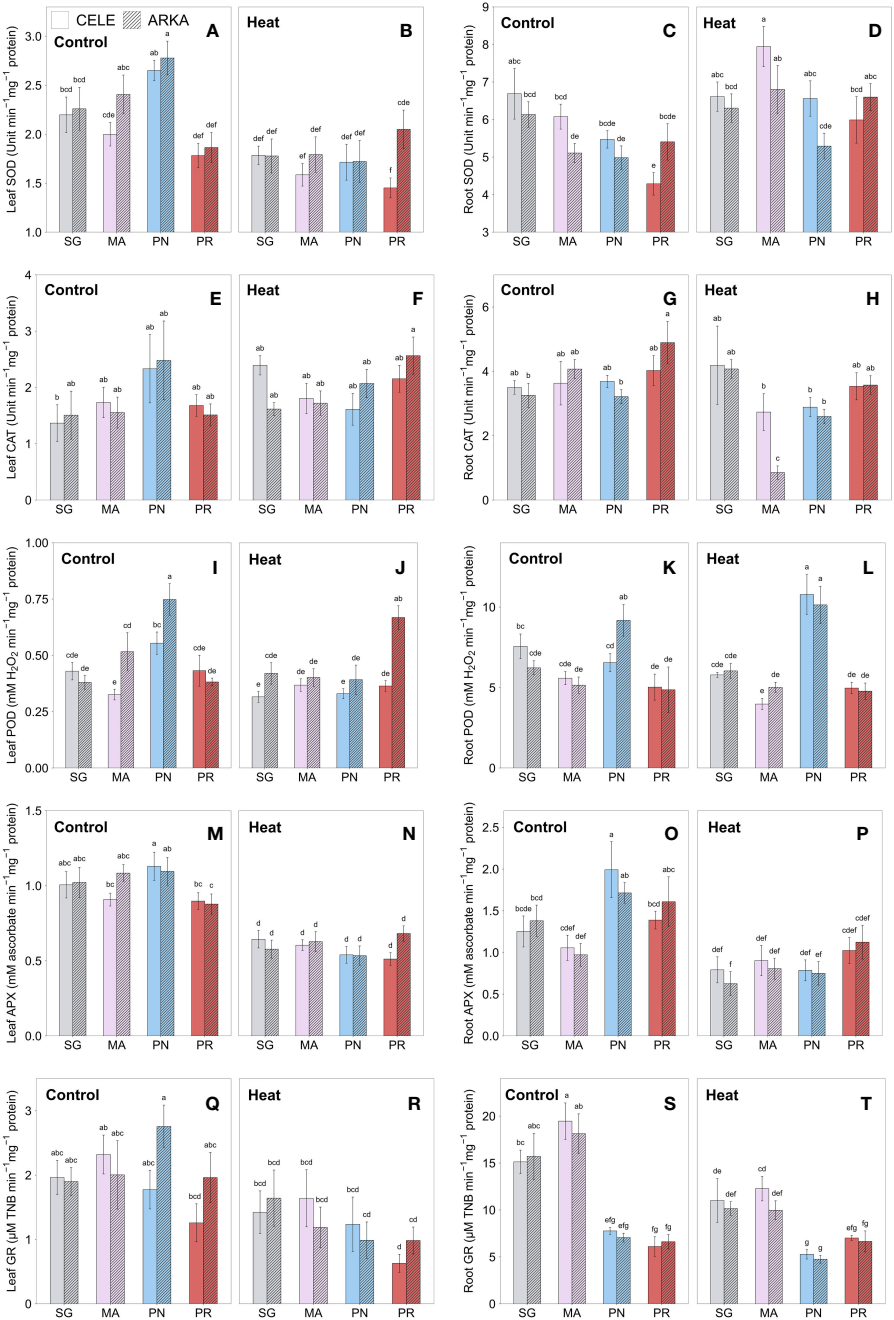
Heat stress responses of leaf SOD **(A, B)**, CAT **(E, F)**, POD **(I, J)**, APX **(M, N)** and GR **(Q, R)**, and root SOD **(C, D)**, CAT **(G, H)**, POD **(K, L)**, APX **(O, P)** and GR **(S, T)** in tomato plants self-grafted or grafted onto ‘Maxifort’, *S. pennellii* and *S. peruvianum* rootstocks. Different letters indicate significant differences among scion-rootstock combinations across the temperature treatments (control, heat) at *P* ≤ 0.05; CELE, ‘Celebrity’; ARKA, ‘Arkansas Traveler’; SG, self-grafted; MA, ‘Maxifort’; PN, *S. pennellii*; PR, *S. peruvianum*.

**Figure 4** shows the rootstock and scion cultivar effects on antioxidant enzyme activities in leaves and roots after three weeks of heat stress conditions. Leaf SOD activities were significantly reduced by heat stress in MA-grafted ‘Arkansas Traveler’ and PN-grafted plants (**Figures 4A, B**; **Supplementary Figures 1C, D**). PN-grafted plants had a higher enzyme activity than SG ‘Arkansas Traveler’, MA-grafted ‘Celebrity’ and PR-grafted plants at the control temperature within the scion treatments, but there were no significant differences between the treatments under the heat stress conditions, with a significant reduction in the enzyme activities in PN-grafted plants (**Supplementary Table 2**). In root samples, the SOD activities were increased by heat stress in MA-grafted plants and PR-grafted ‘Celebrity’ plants, while no significant changes were detected in the rest of the grafting combinations (**Figures 4C, D**; **Supplementary Table 1**; **Supplementary Figures 1E, F**). Under heat stress conditions, MA-grafted ‘Celebrity’ and ‘Arkansas Traveler’ plants had higher root SOD activities than those in PR and PN-grafted plants, respectively.

There were no significant differences in leaf CAT activities between grafting combinations both in control and heat stress treatment (**Figures 4E, F**), while significant temperature-rootstock interactions were observed with higher fold change from control to heat treatment in PR-grafted plants compared to PN-grafted plants (**Supplementary Table 1**; **Supplementary Figures 1C, D**). In root samples, there were no significant differences between treatments in ‘Celebrity’ plants, but in ‘Arkansas Traveler’, PR-grafted plants had a higher CAT activity than SG and PN-grafted plants in control treatment and MA-grafted plants showed the lowest enzyme activity under heat stress (**Figures 4G, H**).

In **Figures 4I, J**, PN and PR-grafted plants showed contrasting heat responses in leaf POD activities. The enzyme activities of PN-grafted ‘Arkansas Traveler’ plants, which were the highest among the treatment groups at control temperature, significantly decreased by heat stress and became similar to those of SG and MA-grafted plants (**Supplementary Table 2**). On the other hand, leaf POD activities of PR-grafted ‘Arkansas Traveler’ plants significantly increased from control to heat treatments (**Supplementary Figure 1D**) and had the highest enzyme activities under heat stress conditions among all grafting combinations. In root POD activities, PN-grafted plants had the highest enzyme activities under heat stress with significant enzyme activity increase from control to heat treatments in ‘Celebrity’ treatment groups, while no significant heat stress responses were observed in other grafting combinations. (**Figures 4K, L**; **Supplementary Figure 1E**).

The APX activities in leaves showed a significant decrease under heat stress in all grafting combinations (**Figures 4M, N**; **Supplementary Table 2**). At control temperature, PN-grafted plants demonstrated significantly higher enzyme activities than MA-grafted ‘Celebrity’ and PR-grafted plants, but they showed the greatest reduction from control to heat stress conditions (**Supplementary Figures 1C, D**). On the other hand, PR-grafted ‘Arkansas Traveler’ showed the lowest decrease among all grafting combinations. A similar trend was found in root samples that PN-grafted plants had the largest reduction in root APX activities among the grafting combinations (**Figures 4O, P**; **Supplementary Figures 1E, F**), despite their significantly higher enzyme activities at control temperature compared to SG and MA-grafted plants.

**Figures 4Q, R** show a significant decrease in leaf GR activities under heat stress conditions only in PN-grafted ‘Arkansas Traveler’, while no other significant differences were observed between grafting combinations within the temperature regime or any interaction effects. Root GR activities were significantly higher in SG and MA-grafted plants than PN and PR-grafted plants in the control treatment, but they showed a greater decrease when exposed to heat stress (**Figures 4S, T**; **Supplementary Figures 1E, F**). Still, the enzyme activities in PN-grafted plants remained significantly lower than SG and MA-grafted plants.

### 3.5 Abscisic acid content

**Figure 5:**
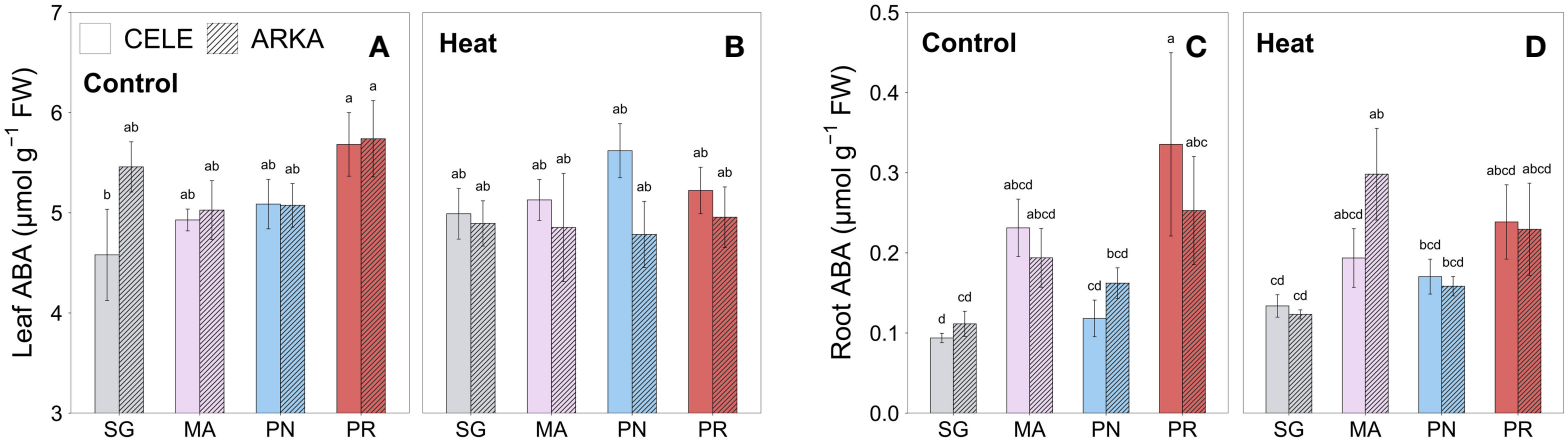
Heat stress responses of leaf **(A, B)** and root **(C, D)** ABA content in tomato plants self-grafted or grafted onto ‘Maxifort’, *S. pennellii* and *S. peruvianum* rootstocks. Different letters indicate significant differences among scion-rootstock combinations across the temperature treatments (control, heat) at *P* ≤ 0.05; CELE, ‘Celebrity’; ARKA, ‘Arkansas Traveler’; SG, self-grafted; MA, ‘Maxifort’; PN, *S. pennellii*; PR, *S. peruvianum*.

The leaf ABA contents were significantly higher in PR-grafted ‘Celebrity’ than that of SG plants in control treatment, but there were no significant differences between the grafting combinations under heat stress conditions (**Figures 5A, B**; **Supplementary Figures 1C, D**). Similar results were observed in root samples that PR-grafted ‘Celebrity’ had significantly higher ABA content than that of SG and PN-grafted plants at control temperature, but the differences were reduced when exposed to heat stress (**Figures 5C, D**; **Supplementary Figures 1E, F**). Under heat stress, MA-grafted ‘Arkansas Traveler’ showed a higher root ABA content than that of SG plants.

## 4 Discussion

### 4.1 Poor growth of PN and PR-grafted plants despite heat-tolerant responses in physiology

Plant biomass is a reliable indicator of overall fitness and reproductive capacity (Younginger et al., 2017), and it is often considered as a degree of heat stress tolerance (Patel and Franklin, 2009; Lipiec et al., 2013; Wassie et al., 2019; Molero et al., 2023). Our results indicated that the shoot growth of PN and PR-grafted plants were significantly lower than SG and MA-grafted plants in both control and heat stress treatments, even though there were no significant differences in net photosynthetic rate, leaf chlorophyll content and chlorophyll fluorescence among the grafting combinations (**Figures 1E, F**, **2A–D**). PN and PR-grafted plants showed several distinctive heat-tolerant physiological responses compared to SG and MA-grafted plants. PN-grafted plants had a greater increase in net photosynthetic rate and leaf chlorophyll content when exposed to heat stress and there were no deleterious stress effects in chlorophyll fluorescence of PN and PR-grafted ‘Arkansas Traveler’ plants (**Figures 2A, B**; **Supplementary Figures 1A, B**), which indicated the negligible amount of oxidative damage and dislodgement of PSII reaction centers from high temperature stress (Baker, 2008). However, the physiological benefits of PN and PR-grafted plants did not lead to an increase in plant growth as they exhibited significantly lower shoot FW compared to SG and MA-grafted plants (**Figures 1C, D**). This is not entirely unexpected, as instantaneous net photosynthetic rates are not predictive of overall plant biomass production, while it is more influenced by nutrient and water uptake availability (Kramer, 1981; Sinclair et al., 2019). Considering that graft incompatibility increases with higher taxonomic distance (Goldschmidt, 2014), the poor growth of the wild relative rootstock-grafted plants could be due to the interspecific grafting combinations of *S. lycopersicum* scion cultivars and wild rootstock species, *S. pennellii* and *S. peruvianum*. Julián et al. (2013) also stated that PR (PI126944) had strong incompatibility with cultivated tomato plants. In our experiment, the grafting success rate of wild relative rootstocks was distinctly lower (~ 50-70%) than that of the commercial MA rootstock (> 85%), a pattern also seen in a tomato grafting study by Zeist et al. (2017) who examined the low graft success rate of PN rootstocks. Graft incompatibility arises from the imperfect connection of the vascular system at the graft union, which causes the inhibition of nutrient and water uptake as well as the root-shoot transport of assimilates (Kawaguchi et al., 2008). These results suggest that graft incompatibility could be the reason for the reduced growth of PN and PR-grafted plants despite their high physiological performance. The smaller stem diameter of PN and PR-grafted plants in both the control and heat stress environment (**Figures 1A, B**) also supports the result that the recovery of the vascular system at the graft union was not as successful as in the SG and MA-grafted plants during the graft healing process.

Several studies have identified physiological characteristics of tomato plants as important indicators of heat tolerance, such as leaf chlorophyll fluorescence, electrolyte leakage and carbohydrate content (Zhou et al., 2015; Zhou et al., 2017; Bhattarai et al., 2021). However, strategies for heat tolerance differ based on species and cultivars (Wahid et al., 2007), and even plants recognized for their high heat tolerance may exhibit several susceptible traits and heat-sensitive plants can have heat tolerance traits. For example, a study by Rajametov et al. (2021) showed that heat-sensitive tomato cultivar had greater root FW and fruit yield than a tolerant cultivar, and Li et al. (2015) also demonstrated a higher level of gene expression in heat shock proteins in a pepper cultivar with greater heat susceptibility. In our study, establishing a clear relationship between heat-tolerant physiological traits and plant growth proved challenging due to graft incompatibility between commercial tomato cultivars and wild relative rootstocks. To identify a significant indicator of heat tolerance in our study, it is necessary to work with scion cultivars that exhibit higher graft compatibility with wild relative rootstocks.

### 4.2 High antioxidant capacity of PR-grafted plants and heat-sensitive enzyme activities of PN-grafted plants

Despite the reduced plant growth due to low graft compatibility, PR-grafted plants showed superior antioxidant capacity among all rootstock treatment groups. The non-enzymatic total antioxidant capacity, measured using a ferric reducing antioxidant power (FRAP) assay, was numerically the highest in PR-grafted plants on average among all treatment groups in leaves, and their root antioxidant capacity was also higher than that of SG and MA-grafted plants under no stress and heat stress conditions (**Figures 3E–H**). This indicates that PR rootstocks have a naturally higher non-enzymatic antioxidant production regardless of the stress conditions compared to other rootstocks and successful root to shoot transport of antioxidants or their signaling molecules (Dodd, 2005). The leaf proline content, which was highly correlated with FRAP results (r = 0.86, *P* < 0.001), contributed to the high antioxidant capacity of PR-grafted plants. Under heat stress conditions, proline works as a compatible osmolyte which stabilizes the antioxidant system and maintains the redox balance and protein native structure (Szabados and Savoure, 2010; Zandalinas et al., 2016). Our study showed that PR-grafted plants had similar or higher leaf proline content than other grafted combinations at both temperature regimes within the scion treatments (**Figures 3I, J**), and also had a greater increase from control to heat treatment compared to SG plants, particularly in ‘Celebrity’ (**Supplementary Figures 1A, B**). According to Wahid et al. (2007) and Iqbal et al. (2019), heat stress responses with larger increases in total antioxidant capacity and leaf proline contents are considered as evidence of heat tolerance. Therefore, the high total antioxidant capacity and leaf proline content in heat-stressed PR-grafted plants indicate that the thermotolerance of tomato plants could be enhanced by grafting with PR rootstocks.

PR-grafted plants also demonstrated heat tolerance in leaf enzymatic antioxidant activities. Their leaf antioxidant enzyme activities at control temperature were similar or lower than other rootstock treatment groups but PR-grafted plants had a smaller magnitude of decrease (**Figures 4A, B, M, N**) or greater increase (**Figures 4E, F, I, J**) in multiple enzyme activities when subjected to heat stress conditions compared to other grafting combinations, especially in the ‘Arkansas Traveler’ scion cultivar (**Supplementary Table 2**; **Supplementary Figures 1C, D**). These results indicated that grafting onto PR rootstocks not only enhanced the non-enzymatic antioxidant capacity but also established a heat-tolerant enzymatic antioxidant system in leaves. Similar results were reported by Rivero et al. (2003b) in which heat-stressed grafted tomato plants showed thermotolerance with a smaller decrease of CAT, POD, APX and GR enzyme activities than non-grafted controls, and Li et al. (2014) who demonstrated that heat-tolerant Kentucky bluegrass cultivar had a greater increase or smaller decrease of SOD, CAT, POD and APX activities than heat-sensitive cultivar during 28 days of heat treatment. CAT, POD and APX are antioxidant enzymes that prevent oxidative cell damage by scavenging H_2_O_2_ (Inze and Van Montagu, 1995). Our results demonstrated that PR-grafted plants made greater use of CAT and POD as H_2_O_2_ scavenging compounds in leaves than the ascorbate-glutathione pathway, which includes APX and GR (Hasanuzzaman et al., 2019), compared to other rootstock treatment groups. However, there were no significant differences in leaf ROS content between PR-grafted plants and other treatment groups, and the correlations between leaf ROS and the enzymatic and non-enzymatic antioxidant activities were also non-significant (r = – 0.04, *P* = 0.371) and very weak (r = 0.27, *P* = 0.012), respectively. Such unclear relationship between ROS and antioxidants could be due to other ROS detoxifying enzyme activities, such as glutathione peroxidase and dehydroascorbate reductase, which were not measured in this study, or non-enzymatic antioxidants that could not fully measured in the FRAP assay due to the limitations of the method (Rubio et al., 2016; Benzie and Devaki, 2018). It is possible that after 21 days of stress period, the plants could already have degraded a significant amount of the leaf ROS content to the tolerable level. However, further studies on plant response to short and long-term heat stress conditions are needed to identify the comprehensive antioxidant-ROS interactions of grafted tomato plants.

On the other hand, PN-grafted plants showed heat-sensitive antioxidant enzyme responses in leaves with a significant reduction of enzyme activities (leaf SOD, POD and APX in both scion cultivars, and GR in ‘Arkansas Traveler’) when the plants were exposed to heat stress conditions (**Supplementary Table 2**; **Supplementary Figures 1C, D**), although they had numerically the highest value at control temperature among the rootstock treatments (**Figure 4**). This indicates that using PN rootstocks could enhance the antioxidant enzyme activities by grafting in a stress-free environment, but the beneficial traits could not be sustained under high temperature conditions. Considering that PN demonstrated higher leaf SOD and APX activities than cultivated tomato plants at both control and salinity stress conditions (Shalata and Tal, 1998), it is speculated that the superior leaf antioxidant enzyme activities of PN-grafted plants at control temperature could be used as a tolerance strategy under other abiotic stress conditions such as drought and salinity. In contrast to the enzymatic antioxidant responses, there were no heat-sensitive responses in leaf and root non-enzymatic antioxidant capacity of PN-grafted plants, rather they showed similar or better results than other treatment groups (**Figures 3E–H**; **Supplementary Figures 1C, D**). Considering the absence of significant differences in leaf ROS contents under heat stress among grafting combinations (**Figures 3A, B**), the overall results suggest that the non-enzymatic antioxidants were used as a primary stress defense mechanism in PN-grafted plants while their enzymatic antioxidant system may not be well adapted for heat stress conditions. This could be due to the metabolic efficiency of PN-grafted plants focusing on the accumulation of non-enzymatic antioxidants rather than the enzymes, or they could be lacking antioxidant enzyme gene expression induced by heat stress compared to other rootstock genotypes (Bita et al., 2011). This is consistent with PN’s drought and salinity stress tolerance strategies which produce large numbers of compatible solutes (Alarcon et al., 1993; Santa-Cruz et al., 1999; Atherton and Rudich, 2012), although PN-grafted plants could not successfully transfer their superior non-enzymatic antioxidant capacity from rootstock to scion (**Figures 3E–H**).

Unlike significant responses of PN and PR-grafted plants in leaves, root antioxidant enzymes were differentially regulated by rootstocks (**Figure 4**). PN-grafted plants had the highest root POD activities under heat stress while their reduction in APX activity was greater than in the other grafting treatment groups (**Supplementary Figures 1E, F**). PR-grafted plants also showed an increase in root SOD and decrease in root APX under heat stress. The selective regulation of root enzymes were also found in Lee et al. (2023) such that ‘Maxifort’ rootstocks had higher root SOD and CAT and lower root POD activities than ungrafted roots under heat stress and Goyal and Asthir (2010) who demonstrated differential heat stress responses of root CAT, POD and APX in five wheat cultivars. The varying root antioxidant enzyme regulations by different rootstock treatments imply their species’ distinctive phenotypic traits under heat stress environment, while the low correlation between leaf and root enzyme activities (r = 0.22, *P* < 0.001) may be due to interactions of the rootstock × scion × environment effects in leaves (Albacete et al., 2015), but only rootstock × environment interactions in the roots. This indicates that when using rootstocks with heat-tolerant antioxidant capacity, their interaction with scion varieties and stress conditions should be considered so that their beneficial traits are successfully communicated to the shoot.

### 4.3 No significant effect of ABA on heat tolerance and leaf cooling

ABA is known to induce the thermotolerance of plants by enhancing antioxidant capacity, a mechanism that is mediated by ROS signaling (Islam et al., 2018). However, except for a weak correlation between ABA content and FRAP in roots (r = 0.43, *P* < 0.001), our study did not show any significant correlations between ABA content and antioxidant capacity parameters or ROS contents. For example, strong leaf antioxidant capacity was observed in PR-grafted plants although the leaf ABA content was decreased by heat stress with marginal significance (*P* = 0.064), and root FRAP was the lowest in MA-grafted plants despite their high root ABA contents under heat stress conditions (**Figures 3E–H**, **5A–D**). The results suggest that ABA may not be the principal factor regulating the antioxidant system in our study, or the seedlings could have been acclimated during the 21 days of heat stress period with stabilized ABA and ROS responses, but further research on ABA-induced heat tolerance pathway is required for identifying the underlying mechanism.

Heat stress promotes the reduction of leaf temperature by opening the stomates and increasing leaf transpiration rate (Sharma et al., 2015), and ABA is widely known as a key hormone that regulates the stomatal opening. However, leaf ABA content in leaves and roots did not reveal rootstock differences when exposed to heat stress (**Figures 5A–D**) but also had no significant correlation with leaf transpiration rate and stomatal conductance. Similar results were found in a growth chamber study by Zandalinas et al. (2016) that two citrus cultivars showed differential heat responses in leaf ABA contents while the transpiration rate was significantly increased in both cultivars. These results suggest that under long-term heat stress exposure, ABA did not provide leaf cooling protection. Kostaki et al. (2020) also reported that high temperature-induced stomatal opening could be due to the autonomous guard cell responses by integrating light and temperature information with stimulation of thermo-sensing protein activities. Thus, it could be speculated that MA rootstock has more sensitive thermo-sensing mechanisms which are transferrable to scion and could effectively regulate the guard cell than the other rootstocks under heat stress conditions.

### 4.4 Tolerant and susceptible heat responses of scion cultivars

Heat-tolerant ‘Celebrity’ had a higher gas exchange rate, leaf chlorophyll contents and a greater non-enzymatic leaf antioxidant capacity compared to heat-sensitive ‘Arkansas Traveler’ under heat stress. ‘Arkansas Traveler’ showed less heat susceptibility in chlorophyll fluorescence, which contradicts with the results from our previous cultivar screening in the growth chamber and open field study (Bhattarai et al., 2021), possibly due to different plant developmental stage, soil type and temperature regimes, but leaf ROS contents were still lower in ‘Celebrity’ under heat stress at a marginally significant level (*P* = 0.083). Significant temperature × scion interactions were observed in leaf ABA contents such that ‘Celebrity’ had either greater magnitude of increase or smaller decrease than ‘Arkansas Traveler’ when exposed to heat stress depending on the rootstocks (**Supplementary Figures 1C, D**). These responses were similar to the research conducted by Li et al. (2014) and Zandalinas et al. (2016) where tolerant cultivars had a greater increase in ABA than the sensitive cultivars under heat stress. The overall cultivar differences suggest that ‘Celebrity’ suffered less oxidative damage from ROS than ‘Arkansas Traveler’ possibly due to its higher non-enzymatic antioxidant content, such as proline and carotenoids (Lee et al., 2023), which might be induced by increased ABA under heat stress. Also, the effective leaf cooling by higher transpiration could have contributed to its thermotolerance. The only significant rootstock × scion × temperature interaction that specifically enhanced the thermotolerance of heat-sensitive ‘Arkansas Traveler’ scion was observed in leaf POD content which exhibited a greater magnitude of increase under heat stress when grafted onto the PR rootstock (**Supplementary Table 1**). However, we also observed a similar trend in other leaf antioxidant enzymes (SOD, CAT, APX) and gas exchange capacity in **Supplementary Figures 1B, D**, although statistically insignificant, suggesting the potential for utilizing PR rootstock to enhance the thermotolerance of heat-sensitive cultivars.

### 4.5 Transferable heat-stress traits of PN and PR rootstocks

The drought tolerant strategy of PN and PR species were identified as the accumulation of large numbers of compatible osmolytes such as total carbohydrates and proline, and high density, sensitive stomata (Atherton and Rudich, 2012; Tapia et al., 2016; Egea et al., 2018). Zhou et al. (2018) also evaluated the heat stress responses of PN and PR species and discovered higher gas exchange capacity and chlorophyll fluorescence than cultivated tomato plants. In our study, using PN and PR rootstocks under heat stress did not enhance the leaf transpiration rate of the scion plants, but increased the proline content, especially in PR-grafted plants. Also, chlorophyll fluorescence was higher under heat stress in PN and PR-grafted plants compared to SG plants within each scion cultivar (**Figures 2A, B**), and they had a greater increase in net photosynthetic rates under heat stress (**Supplementary Figures 1A, B**). These results indicate that the beneficial stress-tolerance traits of PN and PR species could be transferred to scion plants at a considerable level when they are used as rootstocks.

The mechanisms responsible for transferring the thermotolerance traits from rootstock to scion were not investigated in this study, but it is hypothesized that stress response molecules, including mRNAs, small RNAs (siRNA, miRNA) and proteins such as transcription factors and RNA-binding proteins, produced in rootstock tissues, may be transported to scions (Kondhare et al., 2021). These molecules can be transported through plasmodesmata for short distances (cell-to-cell, cell-to-phloem) and through the phloem for long distances (Kehr and Buhtz, 2008). The transported molecules, which could function as long-distance signals, have the potential to alter gene expression and regulate the epigenetic modifications by DNA and histone methylation in scion tissues in response to stress conditions (Tsaballa et al., 2021). The thermotolerance traits observed in PN- and PR-grafted plants that corresponded with the natural stress response strategies of PN and PR species suggest that the molecules regulating those responses were successfully transported from rootstock to scion. However, our study has limitations in analyzing these mechanisms, and a follow-up study with phloem and xylem sap analysis is required to identify the specific signaling molecules transported from PN and PR rootstocks that enhanced the thermotolerance of scions, while the performance of transported molecules in scion tissues could still highly depend on the molecular interactions between specific combinations of rootstocks and scions. In addition, it is expected that accessions of diverse tomato wild relatives could respond differently to stress conditions (Zhou et al., 2018), therefore confirmation of plant responses is needed for better selection of accession lines.

## 5 Conclusion

This study demonstrated that grafting tomato plants onto *S. peruvianum* (PR) rootstocks enhanced both enzymatic and non-enzymatic antioxidant capacity under heat stress. Conversely, *S. pennellii* (PN)-grafted plants exhibited heat tolerance based on their content of root total antioxidant capacity and leaf proline but demonstrated susceptibility in their antioxidant enzyme activity when plants were exposed to heat stress. Although PN and PR-grafted plants had heat-tolerant responses based on photosynthesis and chlorophyll fluorescence, their growth (shoot fresh weight, stem diameter) were lower than self-grafted and ‘Maxifort’-grafted plants regardless of the stress conditions, possibly due to low graft compatibility with scion cultivars. Overall, our results suggest that grafting onto wild relative rootstocks, particularly PR species, has the potential for enhancing the thermotolerance of scion tomato plants during the vegetative stage. Even though this study was conducted with a limited number of accessions, the results could be useful for breeders in developing heat-tolerant interspecific rootstocks and with additional emphasis on enhancing graft compatibility between rootstock and scion.

## Data availability statement

The original contributions presented in the study are included in the article/**Supplementary Material**. Further inquiries can be directed to the corresponding author.

## Author contributions

CL and DL contributed to conceptualization and methodology. CL, JH, and AN implemented the experiment. CL, JH, AN, and KQ conducted the formal analysis. CL wrote the original draft of the manuscript. DL, JH, AN, and KQ conducted a review and editing of the manuscript. DL supervised, provided guidance and acquired the funding. All authors contributed to the article and approved the submitted version.
