# Supplementary material for: Thermotolerance of tomato plants grafted onto wild relative rootstocks
Source: Front Plant Sci. 2023 Nov 20;14:1252456. doi: 10.3389/fpls.2023.1252456 (PMC10694270; doi:10.3389/fpls.2023.1252456)
Supplement: Supplementary file 1 [file DataSheet_1.docx]

Supplementary Material

**Supplementary Table 1.** ANOVA table of measured parameters as influenced by temperature (T), scion cultivar (S), rootstock (R) and their interactions. Time was used as a covariate for gas exchange measurements. SD, stem diameter; FW, shoot fresh weight; Pn, net photosynthetic rate; Cond, stomatal conductance; Trans, transpiration rate; L, leaf; R, root; ROS, reactive oxygen species; FRAP, ferric reducing antioxidant power; SOD, superoxide dismutase; CAT, catalase; POD, guaiacol peroxidase; APX, ascorbate peroxidase; GR, glutathione reductase; ABA, abscisic acid.

|  | SD | FW | SPAD | F_v_/F_m_ | Pn | Cond | Trans | L-ROS | R-ROS | L-FRAP | R-FRAP | Proline |
| --- | --- | --- | --- | --- | --- | --- | --- | --- | --- | --- | --- | --- |
| T | *** | NS | *** | * | *** | *** | *** | *** | ** | *** | NS | *** |
| S | † | * | *** | ** | ** | * | * | NS | NS | *** | NS | *** |
| R | *** | *** | *** | NS | NS | *** | *** | NS | NS | *** | *** | ** |
| T×S | NS | NS | † | * | NS | ** | * | NS | NS | * | NS | *** |
| T×R | NS | NS | *** | NS | NS | NS | NS | NS | NS | NS | * | * |
| S×R | † | NS | NS | NS | NS | NS | NS | NS | NS | NS | NS | † |
| T×S×R | NS | NS | ** | NS | NS | NS | NS | NS | NS | NS | NS | † |
|  |  |  |  |  |  |  |  |  |  |  |  |  |
|  | L-SOD | R-SOD | L-CAT | R-CAT | L-POD | R-POD | L-APX | R-APX | L-GR | R-GR | L-ABA | R-ABA |
| T | *** | *** | NS | ** | ** | NS | *** | *** | *** | *** | NS | NS |
| S | * | † | NS | NS | *** | NS | NS | NS | NS | NS | NS | NS |
| R | ** | ** | NS | ** | ** | *** | NS | ** | † | *** | NS | *** |
| T×S | NS | NS | NS | NS | NS | NS | NS | NS | NS | NS | * | NS |
| T×R | ** | * | * | ** | *** | ** | * | ** | NS | *** | NS | NS |
| S×R | NS | * | NS | NS | NS | NS | NS | NS | NS | NS | NS | NS |
| T×S×R | NS | NS | NS | NS | *** | † | NS | NS | NS | NS | NS | NS |

†, *, ** and *** show significant differences at *P* ≤ 0.1, 0.05, 0.01, and 0.001, respectively; NS, not significant at *P* ≤ 0.1.

**Supplementary Table 2.** Summary of heat stress responses of rootstock and scion treatment groups. Upward (↑) and downward (↓) arrows indicate increased and decreased mean values, respectively, under heat stress conditions compared to the control temperature at a significant level (*P* ≤ 0.05). SD, stem diameter; FW, shoot fresh weight; Pn, net photosynthetic rate; Cond, stomatal conductance; Trans, transpiration rate; L, leaf; R, root; ROS, reactive oxygen species; FRAP, ferric reducing antioxidant power; SOD, superoxide dismutase; CAT, catalase; POD, guaiacol peroxidase; APX, ascorbate peroxidase; GR, glutathione reductase; ABA, abscisic acid.

| Treatment | | SD | FW | SPAD | F_v_/F_m_ | Pn | Cond | Trans | L-ROS | R-ROS | L-FRAP | R-FRAP | Proline |
| --- | --- | --- | --- | --- | --- | --- | --- | --- | --- | --- | --- | --- | --- |
| SG | CELE | − | − | ↑ | ↓ | ↑ | ↑ | ↑ | − | − | ↑ | ↑ | ↑ |
|  | ARKA | − | − | ↑ | − | − | ↑ | ↑ | − | − | ↑ | ↑ | ↑ |
| MA | CELE | ↑ | − | ↑ | − | ↑ | ↑ | ↑ | − | − | ↑ | − | ↑ |
|  | ARKA | ↑ | − | ↑ | − | ↑ | ↑ | ↑ | − | − | ↑ | − | ↑ |
| PN | CELE | − | ↓ | ↑ | − | ↑ | ↑ | ↑ | − | − | ↑ | ↑ | ↑ |
|  | ARKA | ↑ | − | ↑ | − | ↑ | ↑ | ↑ | ↑ | − | ↑ | − | ↑ |
| PR | CELE | − | − | ↑ | − | ↑ | ↑ | ↑ | − | ↓ | ↑ | − | ↑ |
|  | ARKA | − | − | ↑ | − | ↑ | ↑ | ↑ | ↑ | − | ↑ | − | ↑ |
|  |  |  |  |  |  |  |  |  |  |  |  |  |  |
| Treatment | | L-SOD | R-SOD | L-CAT | R-CAT | L-POD | R-POD | L-APX | R-APX | L-GR | R-GR | L-ABA | R-ABA |
| SG | CELE | − | − | ↑ | − | ↓ | − | ↓ | − | − | − | − | ↑ |
|  | ARKA | − | − | − | − | − | − | ↓ | ↓ | − | − | − | − |
| MA | CELE | ↓ | ↑ | − | − | − | ↓ | ↓ | − | − | ↓ | − | − |
|  | ARKA | − | ↑ | − | ↓ | − | − | ↓ | − | − | ↓ | − | − |
| PN | CELE | ↓ | − | − | − | ↓ | ↑ | ↓ | ↓ | − | ↓ | − | − |
|  | ARKA | ↓ | − | − | − | ↓ | − | ↓ | ↓ | ↓ | ↓ | − | − |
| PR | CELE | − | ↑ | − | − | − | − | ↓ | − | − | − | − | − |
|  | ARKA | − | − | ↑ | − | ↑ | − | ↓ | − | − | − | − | − |

**CELE**

**ARKA**


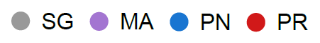

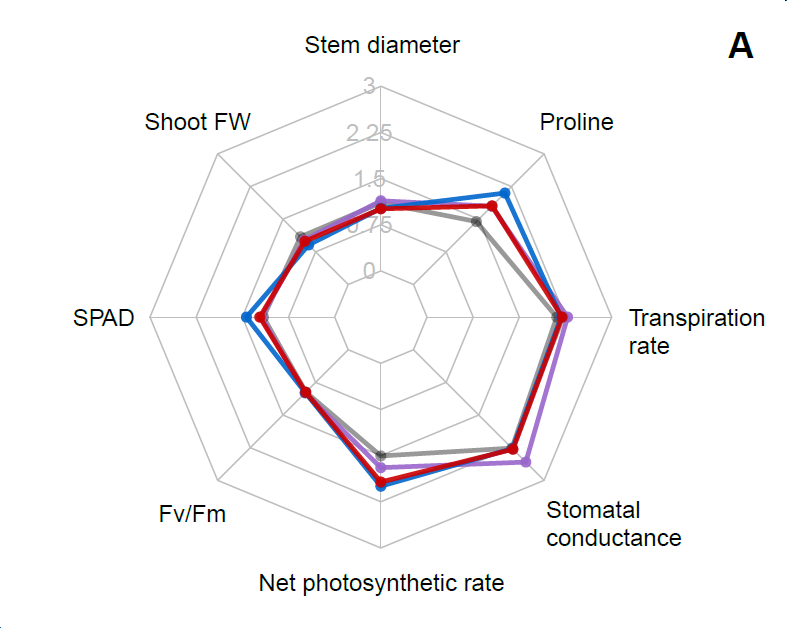

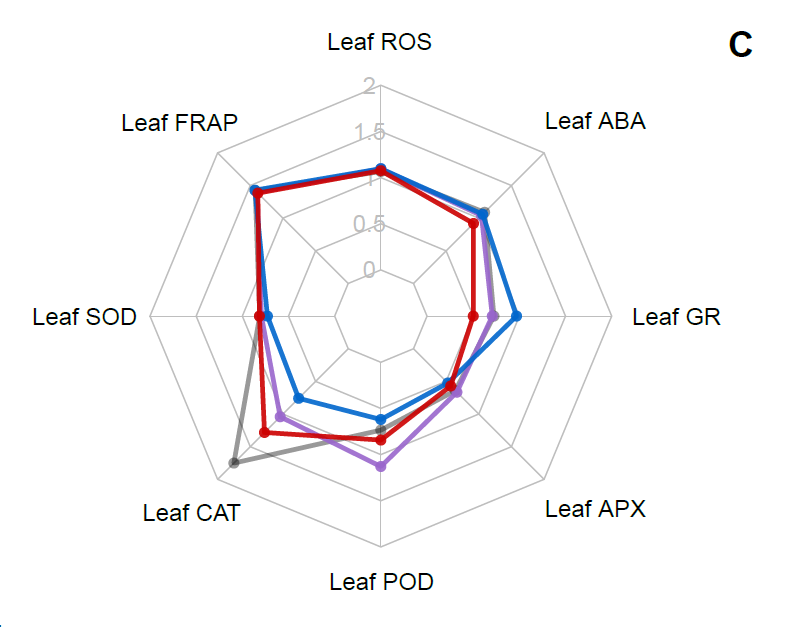

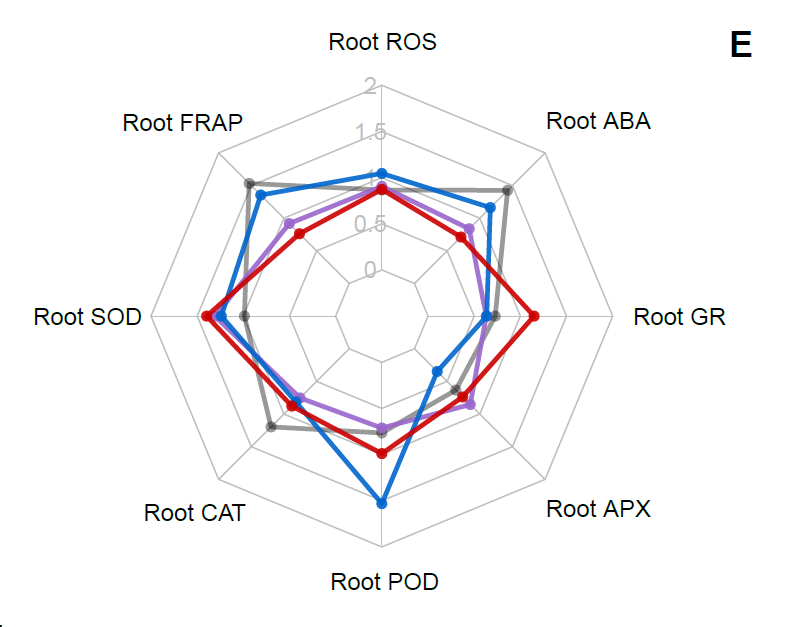

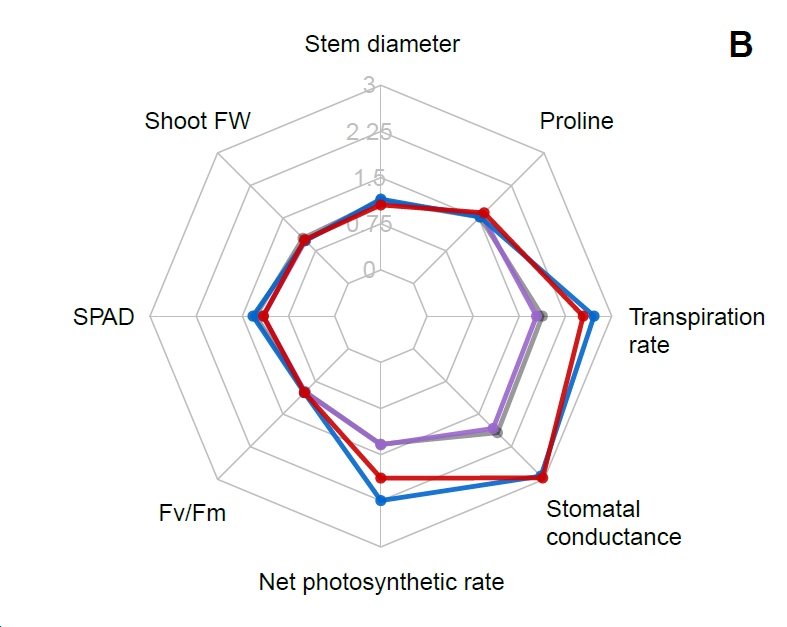

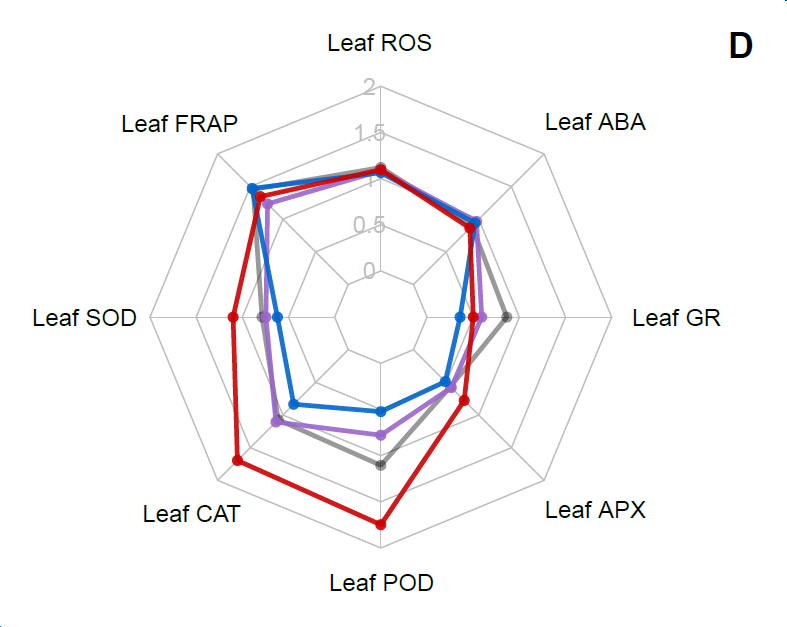

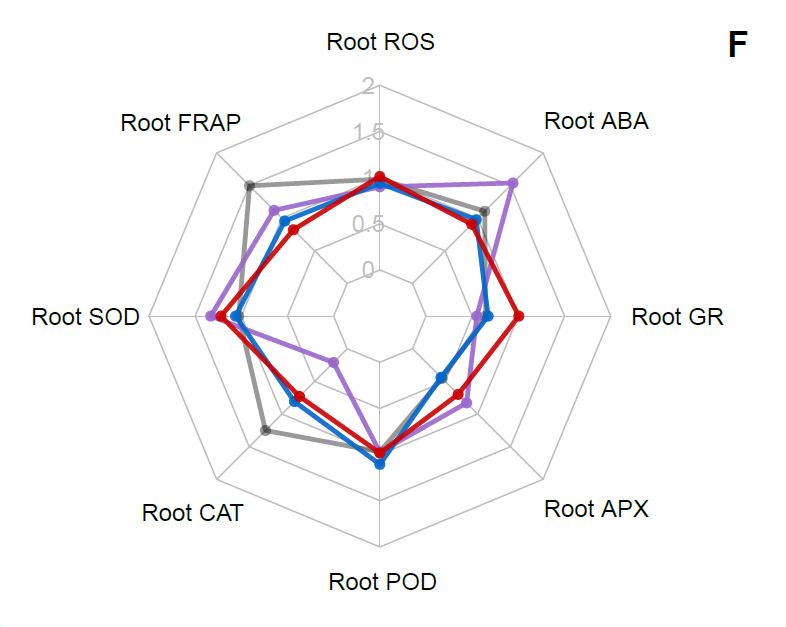


**Supplementary Figure 1.** Fold change of tomato plant growth, physiological and biochemical measurements in heat stress treatments compared to the controls in ‘Celebrity’ (A, C, E) and ‘Arkansas Traveler’ (B, D, F) tomato plants self-grafted or grafted onto ‘Maxifort’, *S. pennellii* and *S. peruvianum* rootstocks; CELE, ‘Celebrity’; ARKA, ‘Arkansas Traveler’; SG, self-grafted; MA, ‘Maxifort’; PN, *S. pennellii*; PR, *S. peruvianum*.
